# Supplementary material for: Identification and functional analysis of non-coding regulatory small RNA FenSr3 in Bacillus amyloliquefaciens LPB-18
Source: PeerJ. 2023 May 15;11:e15236. doi: 10.7717/peerj.15236 (PMC10194069; doi:10.7717/peerj.15236)
Supplement: Supplemental Information 4 [file peerj-11-15236-s004.zip › KO/CK-vs-T1_map/map03440.html]

KEGG PATHWAY: Homologous recombination - Reference pathway


|  |  |
| --- | --- |
| **Homologous recombination - Reference pathway** |  |

[
Pathway menu
| Organism menu
| Pathway entry
| Show description
| User data mapping
]

Homologous recombination (HR) is essential for the accurate repair of DNA double-strand breaks (DSBs), potentially lethal lesions. HR takes place in the late S-G2 phase of the cell cycle and involves the generation of a single-stranded region of DNA, followed by strand invasion, formation of a Holliday junction, DNA synthesis using the intact strand as a template, branch migration and resolution. It is investigated that RecA/Rad51 family proteins play a central role. The breast cancer susceptibility protein Brca2 and the RecQ helicase BLM (Bloom syndrome mutated) are tumor suppressors that maintain genome integrity, at least in part, through HR.

|  |  |  |
| --- | --- | --- |
| Reference pathway | 184% 150% 122% 100% 82% 67% 55% | 图片下载 |
